# High levels of multiple paternity in a spermcast mating freshwater mussel

**DOI:** 10.1002/ece3.4201

**Published:** 2018-07-22

**Authors:** Sebastian Wacker, Bjørn Mejdell Larsen, Per Jakobsen, Sten Karlsson

**Affiliations:** ^1^ Norwegian Institute for Nature Research (NINA) Trondheim Norway; ^2^ Department of Biology University of Bergen Bergen Norway

**Keywords:** bivalves, broadcast spawning, genetic mating system, host‐parasite, Unionidae

## Abstract

Multiple paternity is an important characteristic of the genetic mating system and common across a wide range of taxa. Multiple paternity can increase within‐population genotypic diversity, allowing selection to act on a wider spectre of genotypes, and potentially increasing effective population size. While the genetic mating system has been studied in many species with active mating behavior, little is known about multiple paternity in sessile species releasing gametes into the water. In freshwater mussels, males release sperm into the water, while eggs are retained and fertilized inside the female (spermcast mating). Mature parasitic glochidia are released into the water and attach to the gills of fish where they are encapsulated until settling in the bottom substrate. We used 15 microsatellite markers to detect multiple paternity in a wild population of the freshwater pearl mussel (*Margaritifera margaritifera*). We found multiple paternity in all clutches for which more than two offspring were genotyped, and numbers of sires were extremely high. Thirty‐two sires had contributed to the largest clutch (43 offspring sampled). This study provides the first evidence of multiple paternity in the freshwater pearl mussel, a species that has experienced dramatic declines across Europe. Previous studies on other species of freshwater mussels have detected much lower numbers of sires. Multiple paternity in freshwater pearl mussels may be central for maintaining genetic variability in small and fragmented populations and for their potential to recover after habitat restoration and may also be important in the evolutionary arms race with their fish host with a much shorter generation time.

## 1 INTRODUCTION

Females of many species produce offspring with multiple males within a single reproductive event. Multiple paternity has primarily been studied in mobile species with distinct mating behavior, such as copulation, and typically internal fertilization (Bishop & Pemberton, 1997; Levitan, 2005). Numerous studies in such species have revealed that multiple paternity is highly common in both vertebrates (fish: Coleman & Jones, 2011; amphibians: Adams, Jones, & Arnold, 2005; reptiles: Uller & Olsson, 2008; birds: Griffith, Owens, & Thuman, 2002; mammals: Avise, Tatarenkov, & Liu, 2011) and invertebrates (e.g., crustaceans: Walker, Porter, & Avise, 2002; insects: Simmons, Beveridge, & Kennington, 2007). In contrast, few studies have examined multiple paternity in aquatic species with limited mobility (sedentary or sessile adults) and passive transport of sperm. This mode of reproduction is found in a range of species‐rich benthic marine invertebrate taxa, including ascidians, bryozoans, cnidarians, corals, molluscs, polychaetes, and sponges (Bishop, 1998; Levitan, 1998; Pemberton, Hughes, Manriquez, & Bishop, 2003). Two modes of spawning with passive transport of sperm are commonly distinguished; in broadcast spawning, both sperm and eggs are released, and fertilization takes place in the water column. In spermcast mating, sperm is released into the water, while eggs are retained, and fertilization takes place inside or upon the female (Bishop & Pemberton, 2006).

The mechanisms leading to multiple paternity differ between sessile species with passive transport of sperm and mobile species with distinct mating behavior (Beekman, Nieuwenhuis, Ortiz‐Barrientos, & Evans, 2016; Bishop & Pemberton, 1997). In mobile species, females often actively seek multiple matings. While a single mating would typically suffice to fertilize all eggs of a reproductive event (Bateman, 1948), females may receive other direct (e.g., transfer of resources) or indirect (e.g., increased genetic quality of offspring) benefits from mating multiply (Jennions & Petrie, 2000). In contrast, large brood sizes and passive transport of sperm are expected to result in “obligate” multiple paternity in broadcast spawners and sperm‐casters (Bishop & Pemberton, 1997). Those modes of mating are characterized by random processes, but little is known about the importance of sexual selection via sperm competition and cryptic female choice (Beekman et al., 2016). Nonetheless, while processes of sexual selection may result in nonrandom shares of paternity, the numerous eggs of female broadcast spawners and sperm‐casters are most likely fertilized by the sperm of multiple males (Beekman et al., 2016). In a broadcast spawning sea urchin, for example, between half to all males within a spatial aggregation can fertilize the eggs of a single female (Levitan, 2005).

The genetic mating system describes the distribution of the number of mates among males and females, such as multiple paternity, and can have severe impact on within‐population gene flow and genetic variability. Multiple paternity is expected to decrease variation in male reproductive success and to increase the proportion of reproducing males in the population (Pearse & Anderson, 2009). Populations with higher levels of multiple paternity can thereby maintain higher genetic variability and a larger effective population size (Pearse & Anderson, 2009; Sugg & Chesser, 1994). This can be an important factor for the viability of small populations, where multiple paternity can significantly reduce the likelihood of inbreeding (Mäkinen, Panova, & André, 2007; Moran & Garcia‐Vazquez, 1998). Knowledge on the genetic mating system can thus be important for the estimation of population viability, which is especially important when designing conservation programs for threatened species.

**Figure 1:**
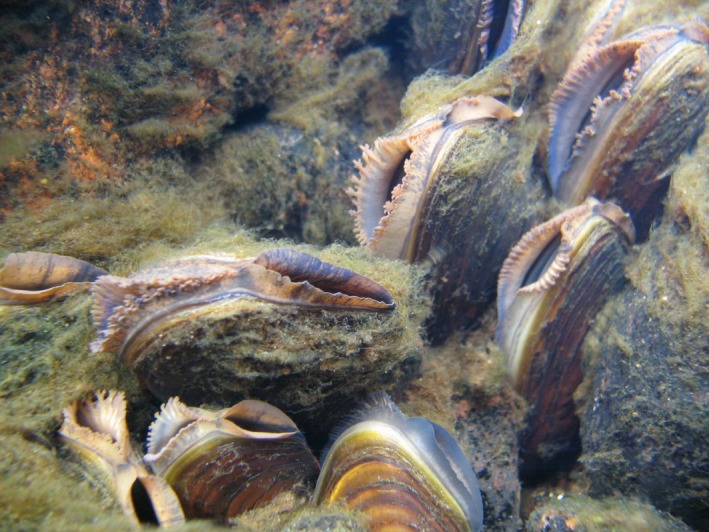
Adult river pearl mussels (*Margaritifera margaritifera*) with syphons exposed

In the present study, we quantified multiple paternity in a natural population of the freshwater pearl mussel (*Margaritifera margaritifera*; Figure 1). As with many species of freshwater mussels (Unionidae), *M. margaritifera* has recently experienced dramatic declines across its holartic range (Lopes‐Lima et al., 2017; Young, Cosgrove, & Hastie, 2001). Many of the remaining populations are today small and exhibit little to no recruitment (Lopes‐Lima et al., 2017; Young et al., 2001). Greater understanding of the reproductive biology could aid in the conservation and management of remaining freshwater pearl mussel populations (Ferguson, Blum, Raymer, Eackles, & Krane, 2013). Freshwater mussels are sperm‐casters, and females capture sperm with their incurrent aperture (Young & Williams, 1984). While reproductive success in female broadcast spawners can be strongly sperm‐limited (Levitan & Young, 1995), sperm limitation may be less important in filter feeding sperm‐casters, which are able to concentrate sperm from low densities (Bishop & Pemberton, 2006; Ferguson et al., 2013; Levitan, 2005). This, together with sperm storage and long‐distance transport of sperm, may make them less vulnerable to Allee effects than broadcast spawners (Ferguson et al., 2013; Mosley, Haag, & Stoeckel, 2014). These conditions may collectively allow females to obtain sperm from multiple males and thereby promote multiple paternity. So far, evidence of moderate levels of multiple paternity has been found in few species of freshwater mussels (*Villosa iris*: Christian, Monroe, Asher, Loutsch, & Berg, 2007; *Lampsilis cardium*: Ferguson et al., 2013; *Hyriopsis cumingii*: Bai et al., 2012). We tested the hypothesis that freshwater pearl mussels also exhibit multiple paternity. We did so using 15 microsatellite markers to assign offspring to mothers and to reconstruct paternal genotypes and thereby the number of sires contributing to each female brood.

## 2 METHODS

### 2.1 Study species

*Margaritifera margaritifera* has a complex reproductive biology that involves an obligatory parasitic stage on a fish host. In summer (June–August in Northern Europe), males release sperm, which females concentrate from the water with their filter feeding system. Eggs are fertilized and developed in a pouch on the gills (Young & Williams, 1984). After ca 4 weeks of development, larvae are released into the water (Hastie & Young, 2003). Larvae are inhaled by a fish host and attach themselves to the gills, where they are encapsulated (Meyers & Millemann, 1977). After 9–11 months, juvenile mussels drop off the gills and settle in the river bed. Juvenile mussels spend 4–8 years buried in the river bed substrate, before they move to the upper parts where they typically have their siphons exposed to filter in open water. In Northern Europe, maturity is reached at an age of 10–15 years and a size of 50–70 mm (shell length) (Bauer, 1987; Lopes‐Lima et al., 2017).

This study was conducted in relation to a captive rearing program of *M. margaritifera* in Norway. Norway holds the largest part of the remaining viable *M. margaritifera* populations in Europe (Larsen, 2010; Lopes‐Lima et al., 2017). At the same time, many populations suffer from reduced or absent recruitment (Larsen, 2010). As part of the national rearing program, adult mussels are collected from natural populations and bred in a hatchery facility. Juveniles are reared beyond the most critical stage of their life cycle and then reintroduced to the original populations.

### 2.2 Collection of adults

A total of 52 adult mussels were collected in Slørdal river (Trøndelag county, Norway) on 20 August 2015, without knowledge of sex and reproductive status (i.e., whether females were gravid), but at a time of the year when they were expected to have passed the period of fertilization. The studied population of *M. margaritifera* in Slørdal river is limited to a section of about 1 km in length, consisting of ca. 3,600 adult individuals (Esplund & Julien, 2016). Most adults in the population are large (i.e., older) mussels and small mussels (<50 mm) have only sporadically been found, suggesting poor recent recruitment (Esplund & Julien, 2016). The average density within the inhabited section is about 15 adults per 100 m^2^, but local densities are as high as 10–20 adults per m^2^ (Esplund & Julien, 2016). Sampling took place in two sections (ca. 350 m apart), with 26 mussels collected in each of them. Those sections were within short distance downstream of the areas with the highest mussel densities. This insured that the female mussels collected would have had optimal conditions for fertilization and the potential for multiple paternity. The collected mussels had a length of 97–146 mm (120 ± 12 mm; mean ± *SD*).

### 2.3 Hatchery

Adult mussels were brought to a hatchery for freshwater pearl mussels in Austevoll (Hordaland county, Norway). In the hatchery, mussels were kept together in a 1 × 1 m tank with circulating permanent water flow (25–35 cm/s). The outgoing water was directed into a tank with fish, ensuring the transport of mussel larvae to a suitable host. Suitable host fishes are trout (*Salmo trutta*) and salmon (*Salmo salar*), with strong preference for either species in each Norwegian mussel population (Karlsson, Larsen, & Hindar, 2014). Both trout and salmon were used in the hatchery. Mussels were inspected regularly for the release of larvae. When all gravid mussels had released their larvae, infected salmon and trout were transferred to separate tanks. Juvenile mussels that had detached from the fish were transferred to channels with suitable substrate for further development.

A minimum of 30 of 52 adult mussels from Slørdal river released larvae (glochidia) in the hatchery, between 5 and 21 September 2015. A total of ca. 4,000 juveniles were produced. Calculating back from the dates larvae were released, eggs were likely fertilized between 8 and 24 August, and thus largely or exclusively before collection of adults in the river. At an age of 9 month (3 June 2016) and 1 year (22 September 2016), 100 juveniles were sampled, respectively, for genetic analysis. Juveniles were randomly sampled and individually stored in ethanol. Sampled juveniles had a length of 0.40 ± 0.04 mm (mean ± *SD*; range: 0.28–0.56 mm) in June and 1.14 ± 0.13 mm (range: 0.90–1.53 mm) in September. DNA samples from the 52 collected adult mussels were taken on 22 September 2016 by gently collecting material from the visceral mass with a cotton swab (Karlsson, Larsen, Eriksen, & Hagen, 2013). Cotton swabs were stored in individual tubes containing 600 μl lysis buffer (Qiagen^™^).

### 2.4 Genetic analysis

For juveniles, DNA was extracted from the whole animal and for adults from cotton swabs using dneasy tissue kits (Qiagen). The mussels were genotyped at 15 loci: MarMa3050, MarMa3621, MarMa4277, MarMa4322, MarMa2671, MarMa4143, MarMa5280 (Geist, Rottmann, Schröder, & Kühn, 2003), and Mm2201, Mm2230, Mm2235, Mm2240, Mm2207, Mm2210, Mm2233, Mm2236 (Garlie, 2010). PCR was carried out in two multiplexes (Karlsson, Larsen, Balstad, Eriksen, & Hagen, 2016). The PCR protocol was as follows: 2 μl DNA, 4 μl Qiagen multiplex mastermix, 0.8 μl primermix, and 1.6 μl RNase free water (Karlsson et al., 2016). The PCR was run on a Quattro Cycler (VWR) in the following conditions: denaturation for 15 min at 95°C, followed by 30 cycles of 57°C for 90 s and 72°C for 60 s, and a final step of 60°C for 30 min (Karlsson et al., 2016). The PCR products of each multiplex were visualized separately on an ABI 3130xl DNA analyzer (Applied Biosystems) and sized using GENEMAPPER ver. 3.7 (Applied Biosystems).

### 2.5 Assignment of parentage

Observed and expected heterozygosity were calculated in CERVUS. The probability of excluding an unrelated individual from parentage (given that the genotype of the other parent is unknown), for each locus and for all loci combined, was calculated in CERVUS. The assumptions of Hardy–Weinberg and linkage disequilibrium were tested on adult genotypes in GENEPOP 4.7 (Rousset, 2008). Significance levels for tests of linkage equilibrium were adjusted using Bonferroni correction.

We assigned parentage to sampled adults using the likelihood‐based approach in CERVUS 3.0 (Kalinowski, Taper, & Marshall, 2007). Sampled adults were known to include all mothers and an unknown fraction of fathers, because reproducing females were gravid when collected from the river. In a first step, we assigned parentage to both parents for offspring for which the father was among the sampled adults. In a second step, we assigned maternity for all other offspring. Females of the species may turn into hermaphrodites under certain conditions (Bauer, 1987), and we allowed for self‐fertilization when assigning parentage.

To assign parentage for offspring for which the father had been sampled, we performed a parent pair analysis with unknown sex. Following the approach in CERVUS, we first ran a simulation (10,000 iterations) to determine the critical Delta value (difference in LOD score between first and second most likely candidate pair) for the assignment of parentage. The critical Delta value is affected by the genetic markers, but also by the proportion of candidate parents that had been sampled. We estimated that 18% of the candidate parents had been sampled, based on the assumptions of an even sex ratio among the 52 sampled adults (Bauer, 1987) and 10 candidate fathers per female mussel (26 candidate mothers, 260 candidate fathers). This allowed for high numbers of sires within broods, as multiple females may share candidate fathers. Not all males in the natural population were considered candidate fathers because females can only reproduce with males located upstream, and with a likelihood decreasing with distance. We repeated the analysis with higher (50%) and lower (5%) estimates of the proportion of sampled candidate parents, which resulted in somewhat higher and considerably lower rates of assignment of paternity to sampled males, respectively (results not presented). However, the assignment of paternity to sampled males did not affect our main results, that is, the reconstructed number of sires per clutch (below).

We then performed a maternity analysis with complete sampling of maternal genotypes to assign the remaining offspring to mothers. All parentage analysis in CERVUS was performed with a mistyping rate of 0.01 and a critical Delta value for parentage assignment set for a confidence level of 95%.

The sex of adults was inferred from assigned parentage. Adults that were assigned parentage in maternity analysis only, or in both maternity and parental pair analyses, were classified as females. Adults that were assigned parentage in parental pair analysis only were classified as males. This was unambiguous for almost all adults. However, one parental pair consisted of adults that both had additional offspring assigned in maternity analysis only and should thus be classified as females according to the above rules. One adult in that pair (SL61) had only a single offspring that was assigned in maternity analysis only. That offspring had a Delta value close to the critical value in parental pair analysis, and we assigned parentage to both candidate parents. Adult SL61 was then classified as male. Another parental pair consisted of individuals that both had common offspring only. We arbitrarily classified one of them (SL53) as female.

### 2.6 Reconstruction of paternity

We calculated two measures of multiple paternity. MINSIRES (Eriksson, Mehlig, Panova, Andre, & Johannesson, 2010) was used to determine the minimum number of sires explaining the offspring genotypes within each brood. MINSIRES is capable of determining the minimum number of sires in cases where many males contribute to a single female brood (Eriksson et al., 2010). COLONY (Wang, 2004) was used to estimate the most probable number of sires per brood with a likelihood‐based approach. Female broods and, for COLONY, known paternity were inferred from analysis in CERVUS. For all analyses in COLONY, allelic dropout rate and loci mistyping rate were set to 0.001 and 0.01 respectively, based on estimates in CERVUS.

### 2.7 Estimating uncertainty in analysis

We used simulated data to test the performance of COLONY and CERVUS in assigning parentage, given the microsatellite markers used in this study. Using the COLONY simulation module (Wang, 2013), we generated adult and offspring genotypes based on the observed allele frequencies. The simulated data were identical with real data in the number of offspring per female (see Results). Genotypes were simulated with a genotyping error of 0.1% and 1% failure of genotyping. We simulated a mating system with a high level of multiple paternity and moderate polygyny (i.e., the degree to which males produce offspring with multiple females). In the simulated mating system, most males sired only a single offspring in each female's clutch. We then analyzed the simulated genotype data in CERVUS and COLONY in the same way as we analyzed the observed data (Wang, 2013). Maternity analysis in CERVUS resulted in a low error rate, with <3% of the offspring assigned to a wrong adult. The number of sires estimated by COLONY was correct for all clutches with one exception, where the number of sires was overestimated by one sire.

### 2.8 Estimating the number of sires in a complete clutch

We genotyped 200 juveniles of a total of approximately 4,000 juveniles produced in our study. The total numbers of sires for each female's full clutch are therefore expected to be much higher than the numbers of sires detected in our samples. In order to estimate the total number of sires in a clutch, we simulated the relationship between the total number of sires in the complete clutch and the detected number of sires in the sample. This was carried out by drawing random samples from simulated complete clutches that varied in the number of sires. Simulations were carried out for the three largest clutches. For smaller clutches, confidence intervals became overly large. Details are presented in the Supporting information (Figure S1).

## 3 RESULTS

### 3.1 Microsatellites

Genotyping was highly successful for all loci (98%–99% of individuals), except for locus MarMa4143 (58%). Two offspring were excluded from analysis because genotyping failed. Of the remaining 250 individuals, 138 individuals (55%) were successfully genotyped at all 15 loci, 109 individuals (44%) at 14 loci, two individuals at 13 loci, and one individual at 11 loci.

The number of alleles per locus ranged from 1 to 25, with a median of nine (Table 1). Observed heterozygosity ranged from 0 to 0.94, with a mean of 0.59. The probability of exclusion for all loci combined was greater than 0.99 (Table 1). We did not detect significant deviations from Hardy–Weinberg equilibrium for any locus or significant linkage disequilibrium among any pair of loci. The estimated rates of null alleles were low for all loci (all <0.015).

**Table 1:** Summary statistics of 15 microsatellite loci for 52 adult and 198 juvenile *Margaritifera margaritifera* from Slørdal river, Norway

Locus	N	No. of alleles	H_e_	H_o_	Exclusion probability
MarMa3050	249	7	0.704	0.703	0.286
MarMa3621	250	25	0.838	0.840	0.526
MarMa4277	250	14	0.899	0.940	0.654
MarMa4322	246	4	0.516	0.537	0.133
Mm2201	249	20	0.869	0.847	0.589
Mm2230	250	9	0.823	0.876	0.482
Mm2235	250	11	0.824	0.832	0.477
Mm2240	247	4	0.079	0.081	0.003
MarMa2671	250	2	0.147	0.160	0.011
MarMa4143	146	15	0.633	0.603	0.244
MarMa5280	250	1	0	0	0
Mm2207	249	13	0.773	0.815	0.396
Mm2210	249	12	0.752	0.743	0.384
Mm2233	250	3	0.364	0.372	0.066
Mm2236	249	12	0.561	0.554	0.193
All		9	0.586	0.594	>0.99

*Note*

H_e_: expected heterozygosity; H_o_: observed heterozygosity.

Median and means are given across loci for the number of alleles and measures of heterozygosity, respectively. The probability of exclusion is given for each locus separately and for all loci combined.

### 3.2 Parentage

**Figure 2:**
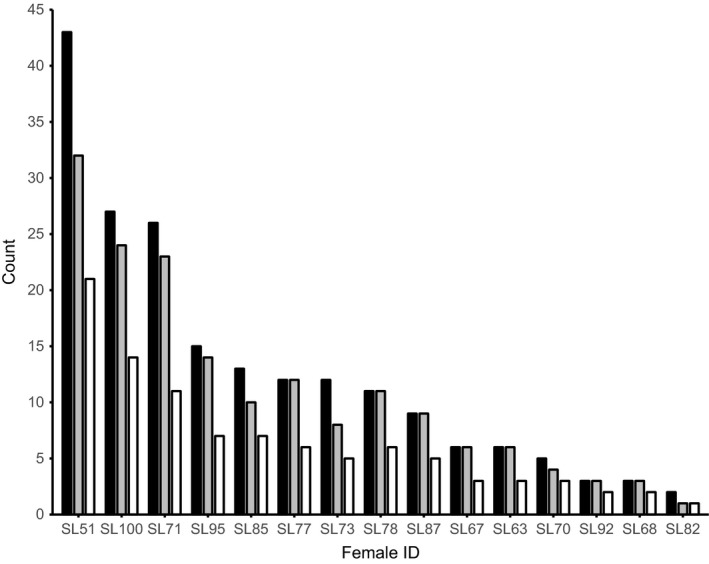
The number of sires in 15 clutches of females from a wild population of *Margaritifera margaritifera*. Black bars show the number of offspring genotyped, gray bars the most likely number of sires (COLONY), and white bars the minimum number of sires (MINSIRES). Not shown are five clutches for which only one offspring was genotyped

A total of 198 offspring were assigned to 20 mothers using CERVUS. The number of offspring assigned to each mother was highly variable (Figure 2) and differed significantly (*df* = 19, χ^2^ = 232.5, *p* < 0.001). The father was detected among the sampled adults for 13 of the genotyped offspring. Those offspring were assigned to seven males, with each male contributing one to five offspring within a brood. Two males sired offspring with two different mothers. We did not detect any cases of self‐fertilization.

### 3.3 Number of sires

The observed extent of multiple paternity was higher than that found in prior studies. We found multiple paternity in all broods for which more than two offspring were genotyped. The most likely number of sires per brood (estimated in COLONY) was close to the number of genotyped offspring in all broods (Figure 2). Thus, most males were estimated to have sired a single genotyped offspring within a brood. In the brood for which 43 offspring were genotyped (SL51), the most likely number of sires estimated by COLONY was 32, and the minimum number of sires detected by MINSIRES was 21 (Figure 2).

We used simulations to estimate the number of sires in the complete broods of the three females for which most offspring were sampled. The complete brood of female SL51 consisted of approximately 870 juveniles, given that 43 offspring were among a sample of 198 juveniles randomly taken from 4,000 juveniles. We used simulations to estimate the number of sires in the complete brood of 870 offspring, given that 32 sires were detected in a sample of 43 offspring. Those simulations suggested that the number of sires in the complete clutch was most likely approximately 68 and between 44 and 127 (Supporting information Figure S1). Applying the same analysis to females SL100 and SL71, most likely numbers of sires were 110 (between 43 and 142) and 103 (between 40 and 321) in the complete broods of 550 and 530 offspring, respectively.

## 4 DISCUSSION

### 4.1 Multiple paternity

We have demonstrated a very high level of multiple paternity in a natural freshwater pearl mussel population. Multiple paternity is expected to increase effective population size and capacity for local adaptation. The freshwater pearl mussel has a parasitic life stage and, contrary to most parasitic species (Gandon & Michalakis, 2002), a much longer generation time than its host (Bauer, 1997). The high level of multiple paternity is likely to be a key feature of the species’ reproductive biology that enables it to maintain and efficiently utilize standing genetic variation in an evolutionary arms race with the fish host. Females may benefit from multiple paternity by increasing genetic diversity among their offspring, which can be considered a form of bet‐hedging. Genetic diversity may be an important determinant of the compatibility and virulence of larvae toward the fish host.

The high degree of multiple paternity in freshwater pearl mussels can be explained by the species’ mode of spawning. Females hold a very large number of eggs (several millions) that are fertilized by males releasing sperm upstream of the females (Bauer, 1987). Given that the sperm of freshwater mussels can be transported efficiently by the water current and may survive for extended periods (Jansen, Bauer, & Zahner‐Meike, 2001; Mosley et al., 2014), several hundred males were potentially within reach of transferring sperm to each female collected in our study. Our results show that either (a) sires of each brood had released sperm within a short time window before fertilization, that (b) females are able to store sperm or that (c) the eggs of one female can be fertilized over an extended period. Further study will be necessary to differentiate among these scenarios, as the timing and synchronization of male spawning and potential storage of sperm are generally poorly understood in freshwater mussels (Ferguson et al., 2013; Hastie & Young, 2003). For example, sperm storage has been found in other sperm‐casters including bivalves (Bishop & Pemberton, 2006; Lutzen, Jespersen, & Russell, 2015), but not for freshwater mussels (Ferguson et al., 2013).

The number of sires was much higher than previously reported for unionid mussels. Between two and four sires were found in broods of other freshwater mussels when 25–29 offspring were sampled (*L. cardium*: Ferguson et al., 2013; *H. cumingii*: Bai et al., 2012). However, the potential for multiple paternity in *L. cardium* was limited by the size of the studied populations (21–41 adults) (Ferguson et al., 2013). Samples of *H. cumingii* were taken from a supposedly large population in a lake (Bai et al., 2012), which raises the possibility that rates of multiple paternity are lower in populations or species inhabiting lentic versus those inhabiting lotic environments. Sperm may be transported more efficiently by the water current of a river than in a lake (Yund, 2000). Our study species shares basic patterns of its reproductive biology with most other species of freshwater mussels. It is therefore likely that high levels of multiple paternity are a common characteristic of the genetic mating system of freshwater mussels.

### 4.2 Consequences for conservation

Evidence of extensive multiple paternity offers new perspectives on reproductive potential that can be leveraged to improve conservation of *M. margaritifera*. Multiple paternity increases the effective population size when compared to monogamous or polygynous mating (Pearse & Anderson, 2009; Sugg & Chesser, 1994). Especially in small populations, multiple paternity can severely reduce inbreeding and thereby maintain genetic variability and population viability (Martinez et al., 2000; Moran & Garcia‐Vazquez, 1998). Many of the remaining populations of *M. margaritifera* are small, making them vulnerable to inbreeding and Allee effects (Geist & Kuehn, 2005). Populations within limited geographical distances, and even within river systems, often exhibit low gene flow and can be genetically highly distinct (Geist & Kuehn, 2005; Karlsson et al., 2014). The conservation of remaining populations, including small and fragmented populations, is therefore a central goal of management efforts (Geist & Kuehn, 2005; Karlsson et al., 2014). Knowledge of critical distances and possible obstacles for sperm to reach female mussels downstream might be crucial for making necessary restorations, especially in fragmented populations. High levels of multiple paternity may help explain why very small populations of *M. margaritifera* can display high genetic variability (Geist & Kuehn, 2005). It also affords insight into why, under low densities, females may become hermaphrodites with the possibility to reproduce by selfing (Bauer, 1987); populations may thus have a critical size or density below which the beneficial effects of multiple paternity are lost.

*Margaritifera margaritifera* is among the most long‐lived invertebrates known, reaching over 200 years in Northern Europe (Lopes‐Lima et al., 2017). Populations may therefore hold high numbers of adult mussels, and nonetheless have little to no recruitment for decades. In central and southern Europe, 95% of the remaining populations are considered functionally extinct due to the lack of recruitment (Lopes‐Lima et al., 2017). Lack of recruitment is mainly caused by anthropogenic eutrophication and consequential loss of oxygen rich substrate, which is needed for the development of juvenile mussels (Geist & Auerswald, 2007). The restoration of rivers is therefore essential for the conservation of the species. Our results, revealing a high degree of multiple paternity, suggest that genetic variability may be efficiently carried over to offspring generations once conditions for recruitment are reestablished. Multiple paternity would be expected to increase capacity for local adaptation, which could improve colonization of newly restored areas (Mäkinen et al., 2007).

Not only do our results offer additional evidence of multiple paternity in unionid mussels, our findings illustrate the potential of genetic methods for estimating demographic parameters, including population size. River ecosystems are highly vulnerable to fragmentation, which can prevent completion of bidirectional migratory life cycles like those exhibited by freshwater mussels (Bórquez & Brante, 2017). Rivers worldwide are subject to a high level of human disturbance and have been experiencing an exceptionally rapid loss of biodiversity (Vörösmarty et al., 2010). Parentage analysis may in the future prove highly useful for inferring population parameters for aquatic species, for which traditional methods are labor intense or unreliable (Bravington, Grewe, & Davies, 2016). For example, close‐kin mark–recapture (CKMR) methods can be used to estimate population size from the number of detected parent–offspring pairs relative to the number of sampled offspring and candidate parents (Bravington et al., 2016). While our sampling regime was not suitable to provide an unbiased population estimate, future studies may develop sampling regimes and statistical population models that allow reliable estimation of population size by CKMR in freshwater mussels.

## 5 CONCLUSIONS

Our results show the potential for high levels of multiple paternity in sperm‐casting freshwater mussels. A better understanding of the genetic mating system is critical to the conservation of those species. Future studies need to show whether multiple paternity is widespread in freshwater mussels and how the degree of multiple paternity depends on the size and structure of populations.

## CONFLICT OF INTEREST

None declared.

## AUTHOR CONTRIBUTIONS

S.K. was the project leader, P.J., S.K. and B.M.L. designed and carried out the study. S.W. performed the parentage analysis and wrote the original manuscript. All authors contributed to revisions.

## DATA ACCESSIBILITY

Adult and juvenile genotypes and R script for simulations of sires in complete clutch are available at the Dryad Digital Repository: https://doi.org/10.5061/dryad.fb2f1v3
