# Supplementary material for: High levels of multiple paternity in a spermcast mating freshwater mussel
Source: Ecol Evol. 2018 Jul 22;8(16):8126–34. doi: 10.1002/ece3.4201 (PMC6145300; doi:10.1002/ece3.4201)
Supplement: Supplementary file 1 [file ECE3-8-8126-s001.docx]

**Supplemental Information for:**

**High levels of multiple paternity in a spermcast mating freshwater mussel**

Sebastian Wacker, Bjørn Mejdell Larsen, Per Jakobsen, Sten Karlsson


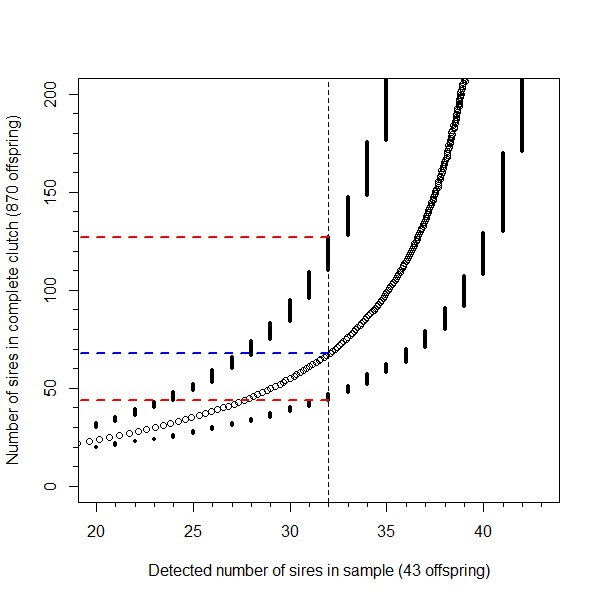


Fig. S1. The simulated relationship between the number of sires detected in a random sample of 43 offspring and the number of sires in the complete clutch of 870 offspring. This is analogous to the largest clutch in our study (female SL51). Open circles show means for each simulation (10,000 iterations) and filled circles show the 95% confidence intervals for each simulation of a given number of sires in the complete clutch. The black dashed line is drawn at 32 detected sires (according to our result for female SL51). Red dashed lines indicate the range of simulations (i.e. number of sires in complete clutch) for which 32 detected sires were within the 95% CI. The blue dashed line indicates the most likely number of sires in the complete clutch, given 32 sires detected in the sample.

Each iteration of a simulation consisted of two steps. First, paternity for the complete clutch was sampled from a Poisson distribution, reflecting equal chances of males to fertilise each offspring. Second, a random sample of 43 offspring was drawn from the complete clutch. The number of sires in the complete clutch was varied by varying the mean of the Poisson distribution.
